# Young Children’s Directed Question Asking in Preschool Classrooms

**DOI:** 10.3390/bs14090754

**Published:** 2024-08-27

**Authors:** Michelle Wong, Koeun Choi, Libby Barak, Elizabeth Lapidow, Jennifer Austin, Patrick Shafto, Elizabeth Bonawitz

**Affiliations:** 1 Graduate School of Education, Harvard University, Cambridge, MA 02138, USA; 2 Department of Human Development and Family Science, Virginia Tech, Blacksburg, VA 24061, USA; 3 Department of Linguistics, Montclair State University, Montclair, NJ 07043, USA; libby.berk@gmail.com; 4 Department of Psychology, University of Waterloo, Waterloo, ON N2L 3G1, Canada; e.lapidow@gmail.com; 5 Department of Spanish and Portuguese Studies, Rutgers University, Newark, NJ 07107, USA; jbaustin@newark.rutgers.edu; 6 Department of Mathematics and Computer Science, Rutgers University, Newark, NJ 07107, USA; patrick.shafto@gmail.com

**Keywords:** preschoolers, question asking, spontaneous speech, social environments, classroom observation

## Abstract

Question asking is a prevalent aspect of children’s speech, providing a means by which young learners can rapidly gain information about the world. Previous research has demonstrated that children exhibit sensitivity to the knowledge state of potential informants in laboratory settings. However, it remains unclear whether and how young children are inclined to direct questions that support learning deeper content to more knowledgeable informants in naturalistic classroom contexts. In this study, we examined children’s question-asking targets (adults, other preschoolers, self-talk) during an open-play period in a US preschool classroom and assessed how the cognitive and linguistic characteristics of questions varied as a function of the intended recipient. Further, we examined how these patterns changed with age. We recorded the spontaneous speech of individual children between the ages of 3 and 6 years (*N* = 30, totaling 2875 utterances) in 40-min open-period sessions in their preschool day, noting whether the speech was directed toward an adult, another child, or was stated to self. We publish this fully transcribed database with contextual and linguistic details coded as open access to all future researchers. We found that questions accounted for a greater proportion of preschoolers’ adult-directed speech than of their child-directed and self-directed speech, with a particular increase in questions that supported broader learning goals when directed to an adult. Younger children directed a higher proportion of learning questions to adults than themselves, whereas older children asked similar proportions of questions to both, suggesting a difference in younger and older children’s question-asking strategies. Although children used greater lexical diversity in questions than in other utterances, their question formulation in terms of length and diversity remained consistent across age and recipient types, reflecting their general linguistic abilities. Our findings reveal that children discriminately choose “what” and “whom” to ask in daily spontaneous conversations. Even in less-structured school contexts, preschoolers direct questions to the informant most likely to be able to provide an adequate answer.

## 1. Introduction

Question asking is a prevalent aspect of children’s speech, providing a means by which young learners can rapidly learn about the world. As such, questions have been a focus of cognitive developmental research for decades [1,2,3,4,5]. Questions support many purposes in childhood, including providing opportunities for conversational clarifications, the acquisition of factual content, and self-reflection, as well as supporting deeper causal–explanatory belief revision (e.g., [6,7,8]). Children’s questions become more selectively and appropriately targeted with increasing age in early childhood; in laboratory studies, it has been demonstrated that preschoolers aged 3–5 years show increasing capability with age in formulating optimal questions to gain information effectively (e.g., [9]) and directing questions with the consideration of the knowledge state of potential informants (e.g., [10]). Similarly, observations of young children’s conversations at home reveal that children seek answers from parents to gain information and follow up when explanations are insufficient, with the frequency of follow-up questions increasing with age [3]. 

Less is known about how questions support the learning of deeper content in pedagogical contexts in which young children spend a considerable amount of their waking hours. Compared to home or laboratory settings, preschool classrooms provide children with a richer social context and opportunities to interact with multiple social partners, including their peers and adults [5,11,12,13]. Despite preschoolers’ increasing ability to selectively determine whose information to trust and whom to ask questions [10,14], formulating questions with proper linguistic characteristics and expressing questions by identifying the appropriate targets can be demanding, particularly in complex social contexts such as the classroom [5]. Although emerging research has begun to examine preschoolers’ spontaneous question asking in preschool classrooms, these studies often focus on questions directed at teachers [15]. Therefore, it remains unclear how preschoolers formulate and express questions in the context of larger social groups, involving both adults and other children, to support their learning goals, despite its significant implications for early childhood education and development. Thus, in this study, we investigated children’s naturally occurring questions during free play time in preschool classrooms, which provide a more realistic context for understanding children’s interactions with others than previously documented in the literature. Specifically, we examined how the cognitive and linguistic characteristics of children’s questions might vary as a function of whom their questions were directed toward and the extent to which these patterns varied by age. 

### 1.1. Preschoolers’ Question Asking

Even the youngest learners play an active, self-directed role in their learning [16]. The benefits of such self-directed learning in early childhood have been supported by a large body of literature on exploratory play and its benefits, such as acquiring strategic skills, setting goals, de-confounding variables, and seeking information [17,18]. Active exploration is considered to be a critical component of learning and an important precursor to scientific reasoning skills, including observation, hypothesis testing via systematic intervention, and data interpretation [19,20,21].

Question asking may provide a means by which children can explore and may provide a particularly powerful way to quickly learn about the world [14,20]. Indeed, question asking is prevalent in young children’s day-to-day conversations [3,6]. In a daily diary study, parents of children between 1 and 5 years old reported children asking on average 76 questions per hour during their conversations with adults at home [3]. The specifics of question-asking behaviors vary across diverse socioeconomic preschool settings in the US [15] as well as cross-culturally (e.g., [1]), but reveal an overall propensity for seeking knowledge via question asking in early childhood. Moreover, prior studies have shown that preschool-aged children utilize questions to gain information from different information sources beyond home or preschool environments, in both research lab and museum settings [22,23].

Although children may ask many questions, there is also evidence that their question-asking strategies are limited by linguistic and cognitive restrictions. Previous studies have examined the potential underlying characteristics and mechanisms of young children’s limited question formulation by examining the linguistic and cognitive features of questions. Some work has suggested that children’s linguistic abilities affect children’s question-asking behaviors, showing a developmental sequence of mastering basic question words that ask for facts or labels (e.g., “what”, “where”, “who” questions) first around age 2, then more open-ended question words (e.g., “why”, “when”, and “how”) afterwards (for review, see [3,24]).

Supporting this progression, by the time children enter preschool, evidence from both lab experiments and naturalistic observations finds that young children use a high percentage of ambiguous questions in these different question types, such as “why?” and “what is this?”, without providing specific details about the object, person, or thing they are asking about, and sometimes with simpler sentence structures, such as “this is real?” (for review, see [22,25,26,27]). Although 4-to-5-year-old children have developed the necessary language skills to ask questions on diverse topics, their questions often remain short and ambiguous [27,28]. As children seek further information, the complexity of their questions tends to increase; a trend that appears to improve with age [9]. For example, Ruggeri et al. (2017) [9] used a question–game paradigm and found that 5 year olds were significantly better than 3 and 4 year olds at identifying the most effective question from a set of options, and that constraint-seeking questions, inquiring about a common characteristic of the group rather than testing a specific hypothesis (“Is it an apple?” instead of “Does it grow on trees?”), only started to emerge around age 6.

Despite their limited linguistic ability, it has been posited that children are increasingly able to make effective queries that gain information in specific ways by knowing what and who to ask in their question expression as they develop throughout early childhood [22,23]. That is, children tailor their questions to request domain-specific information [6,22], and monitor the answers they received to form follow up questions with increasing proficiently as they get older during early childhood [3,7,23]. For example, Nelson et al. (2004) [23] demonstrated that when a child asks “what is it?”, they are more often satisfied with answers that describe a novel object’s function, rather than just the name of the object. Children’s questions become more tailored with age to ask more explanatory and domain-specific information [7,22]. Not only do children actively use questions in their conversations to obtain specific information, but also to seek information from specific people, an ability that improves during preschool years as children grow older. In particular, children extend their question-asking behaviors to become more selective in learning from people who provide accurate information or try to be helpful [29,30,31]. For example, Choi et al. (2018) and Jaswal and Neely (2006) found that children more frequently asked questions intended for learning when they spoke to adults than to the other children, suggesting that children have a propensity to believe that adults have more knowledge than a child [32,33]. Indeed, children become more selective in directing their questions to knowledgeable informants over inaccurate guessers between the ages of 3 and 5 years [10].

To understand the complexities of children’s question-asking behaviors, Ronfard et al. (2018) [5] provide a comprehensive framework detailing the components involved, including question formulation and expression. Formulating a question entails identifying what information to ask for and phrasing the question appropriately. Expressing a question involves determining if there is a reliable source of information available and, if so, assessing whether it is appropriate to request information from them given the context. The components of question formation and expression are posited to be dynamic and work in tandem. That is, children may not only phrase their question and then decide who to ask, but the recipient of their question may also shape how they phrase their questions. Thus, while examining the linguistic characteristics of children’s questions can help us understand how they phrase them, it is crucial to consider what they ask, who they choose to ask, and whether they perceive it appropriate to express in a given social context. Consequently, it is important to analyze both the linguistic features of questions and their association with cognitive characteristics, such as their communicative goal and the intended recipient of the questions posed by preschoolers. Furthermore, the process of question asking is proposed to be supported by cognitive resources, including metacognition, attention, and executive function [5]. These resources develop over time and influence question-asking efficiency, making it integral to examine age-related changes.

### 1.2. Preschoolers’ Question Asking with the Presence of Others

The first aspect of children’s question expression focuses on deciding whether to ask the question, considering the availability and reliability of informants. One reason that even very young children may be able to consider relevant targets for questions is that children are relatively savvy reasoners about others’ knowledge states, intentions, and pedagogical goals, and rely exclusively on informants they deem more “knowledgeable” (e.g., see [29,34]). For example, children draw inferences about the pedagogical goals of teachers from available information to guide their future exploration [35,36,37,38]. When children interpret that a teacher has a goal of sharing pedagogical information, they are more likely to make positive inferences about that teacher. Indeed, the large literature on epistemic trust has documented that children make inferences about sources of information based on the evidence provided to them [39,40,41,42], which includes demonstrating selective learning from those who share the same culture or gender [43,44]. Other, older research has suggested some gender differences in question asking already present in kindergarten years, raising the question of whether gender differences in US children’s question asking might emerge earlier [45].

Furthermore, young children adjust their questions based on how the source of information is characterized [10,28,46]. For example, Mills et al. (2011) [10] presented two puppet informants with varied knowledge states and allowed children aged between 3 and 5 years to freely ask them questions. By age 3, children directed more questions to knowledgeable informants over ignorant ones, suggesting that young children are equipped with a basic skill set to direct questions to the appropriate sources of information. However, there are developmental differences as well. When knowledgeable informants were contrasted with inaccurate guessers, only 5 year olds were able to selectively direct questions to knowledgeable sources, suggesting developmental progression in children’s ability to distinguish between (or at least act on) different knowledge states. These assumptions about the knowledge state of others may shape how children seek out information in the first place, and therefore shape the types of questions they ask and who they are directed toward.

### 1.3. Children’s Question Asking in Preschool Classrooms

The second aspect of children’s question expression focuses on assessing whether it is appropriate to ask a question in a given context [5]. Despite young children’s increasing demonstrations of selectively determining whom to ask questions [10], it remains unclear whether children continue to actively seek out recipients of questions they deem knowledgeable in naturalistic social contexts (such as preschools where seeking information and learning gain is a relevant objective), and how this develops over the preschool years. Constructing questions with appropriate linguistic features and directing them to the appropriate recipients can be demanding, particularly in complex social environments such as classrooms [5].

Several studies have explored children’s questions depending on the knowledge state of the information source (e.g., [10,28,46]). Yet, in these studies, the opportunities for asking questions were driven by the explicit information about the informants and were situated in environments that were structured by researchers; thus, generalization may be limited in what types of behaviors can arise in more natural settings. Recognizing that a fuller picture of question-asking behavior can be enhanced by looking at both the cognitive and linguistic characteristics of children’s questions beyond these controlled settings, we take a naturalistic observational approach to characterize such behaviors and interactions that are more representative of a child’s everyday experience. Therefore, we look beyond the linguistic characteristics of questions and ask how these measures are associated with the communicative goal of the questions preschoolers ask.

Preschools in the United States, importantly, are frequented by children starting at a young age, and are primary educational hubs where children are first introduced to formal instruction. During open activities sessions, preschoolers often have the opportunity to independently work with peers and move throughout the classroom to choose activities and partners. Preschool teachers and aids “float” in the spaces as opportunities for brief one-on-one exchanges. Thus, preschools also differ from home or lab settings by providing children multiple potential conversational partners rather than only a single adult caregiver or experimenter [5,11,12,13].

While opportunities for children to ask questions may vary between cultures, social norms, and educational practices, US preschools often offer open-choice conversational settings, which provide the ideal context to explore how preschoolers differently direct spontaneous questions to different possible partners. Emerging studies have begun to examine young children’s questions in preschool classrooms, showing that children from diverse socioeconomic backgrounds ask similar types of questions to obtain information from teachers, demonstrating the universal role that questions play in their learning [15]. However, these studies primarily focus on children’s questions directed at teachers, rather than exploring how children ask questions beyond teachers, such as peers or themselves. In these contexts, preschool-aged children are developing an ability to identify missing information and ask for it verbally and efficiently, a pivotal milestone not observed in infancy and toddlerhood. At the same early age, a realization of social norms can lead children to gradually adjust their use of questions for learning goals when communicating with adults (e.g., [33,47]). For instance, if preschoolers assume that adults are better sources of information than children [33], and if preschoolers make inferences on an informant’s expertise, we could anticipate children to deem teachers in the classroom as more knowledgeable and children may then ask more tailored information to teachers than they would ask their peers. Therefore, studying how preschool-aged children ask questions in these contexts, with a focus on preschools in the US, is especially vital to understanding early learning and informing early educational practice and how these outcomes are shaped by the specific cultural and social contexts of these schools. Together, these accounts raise rich questions exploring how question-asking behavior develops during the preschool years and how this behavior is manifested in naturalistic contexts.

### 1.4. The Overview of This Study

The objective of this study was to explore children’s naturally occurring questions in a loosely pedagogical context, such as during free play time in US English-speaking preschool classrooms. Of particular interest was to examine any differences in the cognitive and linguistic characteristics of questions as a function of who the question was directed toward and the extent to which these patterns varied by age during preschool years. Compared to home or laboratory settings, preschool classrooms present unique challenges and opportunities to children. We chose to focus on preschool classroom conversations that provide children with a rich social context to interact with multiple social partners including their peers and adults within more formal social norms [5,11,12,13]. The purpose of the current study was twofold. First, we examined whether there are systematic differences in preschoolers’ questions (i.e., quantity, length, and lexical diversity) depending on the goal and target of the conversation. Next, we examined the role of age in children’s question asking (i.e., quantity, length, and lexical diversity), question type, and the recipient of the asked questions.

In the current study, the speech productions of children aged 3 to 6 years were recorded with a device attached to the target child’s clothes, while a live coder noted the contextual information and to whom each individual speech act was directed. While previous work has referenced “wh-” questions as being for learning, this approach may not capture the full richness of learning intent questions (e.g., “What do you mean?” is intended for a person to clarify, “What is that?” is intended for a person to attribute a label to). Thus, we used a novel method to analyze questions for their communicative goal, categorizing them into one of three intents. This allowed us to discern learning-targeted productions from more conversational usages. This extensive coding allowed us to compile a detailed record of questions produced by children as well as the social contexts of the questions during the recording sessions. In addition, we measured question length in words as well as the lexical diversity of utterances (variety and complexity) in order to capture the cognitive and linguistic demands that might limit children’s ability to generate a precise question. Understanding the linguistic characteristics of children’s questions is important because it sheds light on how children formulate and refine their inquiries to seek specific types of information, adapt their language use depending on the social partner, and how their linguistic abilities support their overall inquiry processes [9,22]. This detailed and novel linguistic approach offers a more comprehensive understanding of how such utterances are sensitive to their social environment.

The first goal of the current study was to examine whether preschoolers’ questions (i.e., quantity, type, length, and lexical diversity) vary systematically depending on the goal and target of the conversation. As prior research found that descriptive questions were more common than explanatory questions (e.g., [3]), we hypothesized that the frequency of questions would differ by question type (Hypothesis 1). Based on evidence from prior research on preschoolers’ developing ability to formulate questions [22,23] we expected that children in our sample would present a limited ability to formulate complex and precise questions. While we predict that all question types will include short and ambiguous questions, learning-targeted questions may impose the greatest production challenge for children, reflected by the shorter length of words and lower lexical diversity compared to other question types (Hypothesis 2).

Children’s naturalistic questions in a pedagogical setting like preschools allow us to understand their ability to direct their questions to specific speech partners. We hypothesized that preschoolers in our sample would identify adults (e.g., teachers and classroom aides) as more knowledgeable than other students by directing more questions to adults due to developmental changes in epistemic trust [28,46] and preschoolers’ assumptions that adults are better information sources [33] (Hypothesis 3). Given prior work showing that children produce longer utterances and different words with adult partners than peers [48,49] we hypothesized that the length and lexical diversity of the questions would be greater when directed to adults than to peers or themselves (Hypothesis 4). Furthermore, we hypothesized that adult-directed questions would be more salient among children’s questions intended for learning than other question types (Hypothesis 5), and the length and lexical diversity of the questions would be greater for learning questions directed to adults (Hypothesis 6).

The second goal of the current study was to investigate the effect of age on children’s question asking (i.e., quantity, length, and lexical diversity), question type, and the recipient of asked questions. Given that children’s questions become more tailored with age for more explanatory and domain-specific information [7,22] we specifically hypothesize that one question type, learning questions, in our sample would more likely to reflect the developing ability of children (Hypothesis 7). Based on research with school-aged children, which suggests that their tendency to direct questions to adults may differ in the school environment compared with lab or home settings [13], we hypothesized that there would be age-related differences in the likelihood of children directing questions to adults (Hypothesis 8). Lastly, given the developmental progression of children’s ability to form more specific and advanced questions [3,22,24,25], we hypothesized that the length and lexical diversity of the questions would increase with age (Hypothesis 9).

Although child gender was not a primary focus of our research, previous research suggests that it may influence children’s selective learning and question-asking behaviors [43,44,45]. Thus, we considered gender as a covariate and considered its influence, particularly when its inclusion improved the model fit.

## 2. Methods

### 2.1. Participants

Participants were 30 preschoolers (*M* = 57.8 months, *SD* = 11.1, range = 37–76, 50% female) who participated in their respective childcare classrooms during their free play time. Each classroom was led by a female teacher. The classroom size was an average of 15 children. The sample of participants spanned five classrooms all located in Essex County, NJ, which is one of the most racially and economically diverse counties in the United States as measured by the Gini Index (US Census Bureau, 2016). The number of children per classroom ranged from 2 to 9 (*M* = 6, *SD* = 3.24). We intentionally recruited from these sites to ensure the diversity of the sample. Prior to the study, consent from the sites, participating families, and the internal review board were obtained.

### 2.2. Procedure

Participants were introduced to a clip-on microphone that was attached to the child’s shirt and recorded continuously for the full 40-min session in the child’s classroom. During the session, an experimenter observed surreptitiously from the corner of the classroom. Using time stamps to align with the audio recordings, the experimenter independently recorded both who the participant was speaking to at each moment, as well as every action the participant engaged in during the session. After the session ended, audio recordings were transcribed by another researcher. These transcriptions were linked with the written data using the synced time stamps. This produced a master data set which included the transcribed utterances, contextual cues, and targets of each speech act. Coding and analysis proceeded from the master data set.

### 2.3. Coding

We coded a total of 3619 constructions (*M* = 120.63, *SD* = 78.33 per child, range = 29–352). Of the total constructions, 244 were excluded because of one of the following: non-words (*n* = 18), non-word sound (i.e., screaming or unspellable utterances; *n* = 110), and unidentified constructions (*n* = 116). The remaining 3375 constructions were coded as utterances (*M* = 112.5, *SD* = 74.52 per child, range = 24–349).

For every utterance, we coded whether the speech was directed towards an adult, another child, or self. Of 3375 utterances, 500 were excluded because either the speech partner was unidentifiable (*n* = 471) or child speech was directed to a mixed age group (*n* = 29). The remaining 2875 utterances were included in the analyses (*M* = 95.83, *SD* = 65.29 per child, range: 20–280). Two independent coders coded each of the 2875 utterances. The coders first identified whether each utterance was a question. The interrater reliability between the two independent coders was high; Krippendorff α was 0.93. Discrepancies in codes were reviewed and resolved by a third coder.

Coders were also trained to identify different kinds of questions, dividing questions into one of three subcategories based on the speakers’ intention (see also [50]). (a) Learning questions can be interpreted as intended to learn potentially generalizable content, where learning is broadly defined as acquiring general or specific knowledge about objects, people, or events, including general concepts, rules, or scripts. (b) Communication questions can be interpreted as intended to be rhetorical (giving commands in the form of questions, raising attention with a question) or simply asking for clarification by repeating what others said. (c) Information questions can be interpreted as intended to obtain situation-specific information, such as seeking permission, asking others for an action, checking the physical status of an object (location, belonging, possession), or checking the physical, emotional, or epistemic status of others (other’s needs, opinions). The examples for each question type are included in Table 1. For the three question types, agreement was high; Cohen’s κ was 0.78. Discrepancies in codes were reviewed and resolved by a third coder.

In addition, we used the Stanford CoreNLP toolkit to automatically annotate the utterances [51]. Each utterance was lemmatized, parsed, and part-of-speech tagged. This process provided the number of distinct words used in an utterance, the syntactic pattern, and the number of times each word was used. The length of each utterance in words was calculated as the number of distinct words identified by the toolkit between coded full periods. We omitted words marked as non-legible by the coders, but included names, exclamations, and fillers such as “hmm” and “wow.” Next, we calculated a type–token ratio by dividing the number of distinct words (word types) by the raw number of words (word tokens) to evaluate the informativeness levels of question types. The type–token ratio measures the relative number of words used for each question type, eliminating bias resulting from the differing number of questions observed for each question type. A higher type–token ratio indicates that a larger proportion of the words used are unique, suggesting higher lexical diversity.

### 2.4. Analytical Approach

To test our main research hypotheses, we conducted the following analyses. For the likelihood of asking questions, we created a binary outcome (0: non-question, 1: question) and fitted a mixed effects logistic regression model with subjects as a random effect. For the likelihood of asking specific types of questions (communication, information, learning), we created a binary outcome (0: non-target question, 1: target question) per each question type and fitted a generalized linear mixed effects logistic regression model specified with binomial error structure and logit link function with subjects as a random effect. For average word length and type–token ratio outcome variables, we fitted a mixed effects model predicting each outcome with participant as a random effect. In these models, relevant predictors were included (question type, recipient type, child age) as fixed effects depending on research questions. We included child gender (boy, girl) as a fixed effect into models based on its significance in predicting each outcome variable and the indication of a better fit from the log–likelihood ratio (LLR). These mixed effects were fitted to take into account the dependence of utterances within the same individual child and the varying number of utterances across children [52,53] using either the *lmer* or *glmer* function from the lme4 package in R [54]. This approach allowed us to account for the total number of speech acts directed to each recipient type, ensuring that our analysis controlled for individual differences and the nested structure of the data, providing a robust examination of the factors influencing the propensity to ask questions. Additionally, a Chi-Square Goodness of Fit test was conducted to determine whether the three question types were equally distributed, with ⅓ as the expected proportion.

We note that all data are open access and freely available at: https://osf.io/tdnup/. We include full de-identified transcriptions (combed for removing any personal information) for reproducibility, as well as for supporting future researchers’ novel exploratory analyses.

## 3. Results

### 3.1. Examining the Communicative Goals of Question Asking

**Figure 1:**
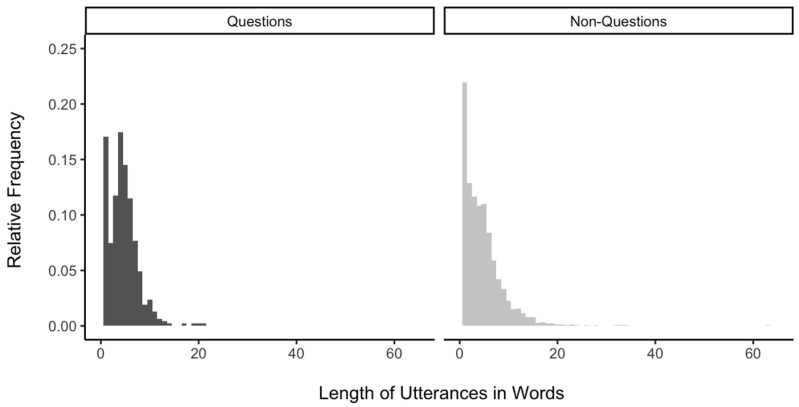
Relative frequency of utterance length in words for questions (on the **right**) and non-questions (on the **left**).

Questions represented 16% of the 2875 coded utterances (*n* = 469; *M* = 16.17, *SD* = 14.5 per child, range: 3–51). The length of words ranged from 1 to 21 words for questions (*M* = 4.59 words, *SD* = 2.94) and 1 to 63 words for non-questions (*M* = 4.72 words, *SD* = 4.07). Girls had shorter lengths of utterances than boys, β = −1.47, *SE* = 0.53, *p* = 0.009. While controlling for the gender effect, the difference between questions and non-questions in the length of utterances in words was not statistically significant, β = −0.04, *SE* = 0.19, *p* = 0.067 (Figure 1). Children appeared to formulate utterances using a comparable number of words, regardless of whether the utterances were questions or not. Additionally, we found that the type–token ratio for questions was higher than that for non-questions, β = 0.04, *SE* = 0.01, *p* < 0.001. Thus, children exhibited higher lexical diversity when asking questions than using other types of utterances. 

We examined whether the frequency of questions varied by the coded categories of questions: communication, information, and learning. A Chi-Square Goodness of Fit test was conducted to determine whether the three question types were equally distributed, with ⅓ as the expected proportion. The proportions significantly differed in the three types of questions, χ^2^(2, *N* = 469) = 100.51, *p* < 0.001. Pairwise comparisons between question types were performed to determine whether the proportions significantly differed from each other, using Chi-Square Tests with the Bonferroni correction. Information questions accounted for the highest percentage (54%, *n* = 252), followed by communication questions (30%, *n* = 140, *p* < 0.001), and then by learning questions (16%, *n* = 77, *p* < 0.001).

**Figure 2:**
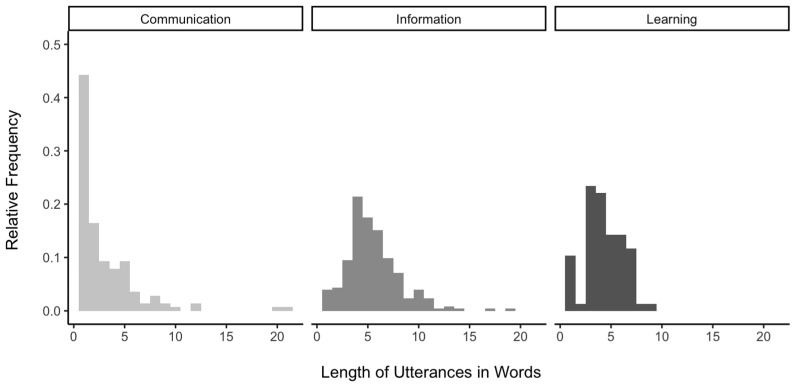
Relative frequency of the length of utterances in words for communication (**left**), information (**middle**), and learning (**right**) questions.

To examine whether the average word length in words varied by question types, we fitted a mixed effects model predicting the length of utterances, with question type and child gender as fixed effects and subject as a random effect. Girls asked questions with shorter lengths in words than boys, β = −0.96, *SE* = 0.45, *p* = 0.045. The length of utterances for communication questions was significantly shorter than that for information questions, β = 2.18, *SE* = 0.29, *p* < 0.001, and learning questions, β = 1.04, *SE* = 0.38, *p* = 0.006. The length of utterances for information questions was higher than learning questions, β = −1.14, *SE* = 0.36, *p* = 0.002. In other words, children used the longest forms to ask information questions (*M* = 5.54, *SD* = 2.68), using many details regarding the subject of the question. Children used mostly short questions for communication goals (*M* = 3.04, *SD* = 3.18) with a high rate of single-word utterances, such as “Huh?” and “What?”. Finally, learning questions (*M* = 4.32, *SD* = 1.86) were longer than communication questions, but ranged over a more narrow length range compared with the information questions with frequent use of similar linguistic formulation, such as “What is this?”. As shown in Figure 2, the distribution of the length of communication questions was right-skewed, primarily influenced by a high proportion of single-word questions. In contrast, information questions included greater length with detailed content, contributing to increased variability. Compared to information questions, learning questions exhibited a narrower distribution, characterized by the frequent use of fixed formulaic questions, used repeatedly with minimal variation.

Next, the lexical variety and complexity of each question type was examined using a type–token ratio. Using a mixed effects model with question type as a fixed effect and subject as a random effect, we found that the type–token ratio did not significantly differ by question type. That is, no significant differences were found between the pairs of question types, namely, learning versus information, β = 0.01, *SE* = 0.01, *p* = 0.279, communication versus information, β = −0.004, *SE* = 0.01, *p* = 0.690, and communication versus learning, β = 0.02, *SE* = 0.01, *p* = 0.187. That is, learning questions contained a comparable number of distinct words to information questions, despite the fact that information questions had a higher word length compared to learning questions, as reported in the previous paragraph. 

### 3.2. Examining the Recipient of Asked Questions

To examine to whom preschoolers directed their questions (adult, peer, and self), a Chi-Square Goodness of Fit test was used. The results showed that the proportions of children’s overall speech (questions and non-questions) significantly differed by recipients, χ^2^(2, *N* = 2890) = 881.8, *p* < 0.001. Pairwise comparisons were conducted to assess the significance of differences in proportions between recipient types using Chi-Square Tests with the Bonferroni correction. Children’s overall speech (questions and non-questions) was directed to other children (59%) more frequently than to themselves (23%, *p* < 0.001) or adults (18%, *p* < 0.001). Further, children’s overall speech was more frequently directed to themselves than adults, *p* = < 0.001. These differences in recipients were likely due to the structure of the free play period with children surrounded by many other children, and only one or two adults in the room.

**Figure 3:**
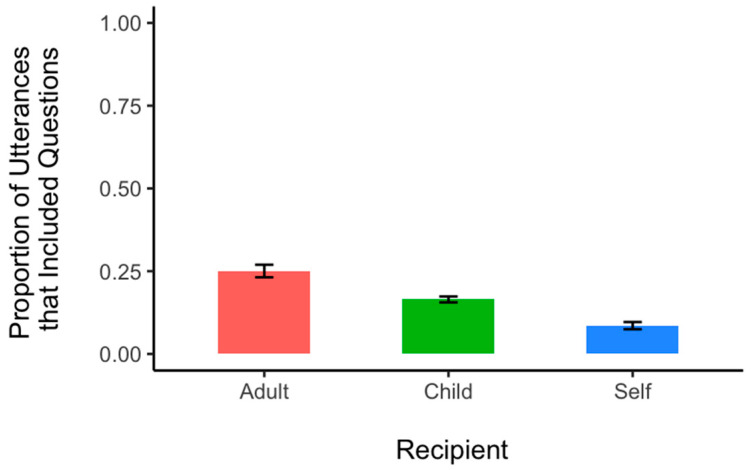
Proportion of children’s utterances including questions as a function of recipient type. The proportion of questions over other possible speech acts based on the recipient of speech production were presented to control for the difference in the amount of speech directed to recipients and uneven child-to-adult ratio in each classroom. That is, the total number of speech acts a child generated for each recipient type was computed first, and then the subset of these acts that were questions were taken as a proportion of that total. Error bars denote *SE*.

To control for the difference in the amount of speech directed to recipients and uneven child-to-adult ratio in each classroom, we fitted a mixed effects model predicting the proportion of questions with recipient type as a fixed effect and subject as a random effect. We found that children were more likely to direct their questions to adults than to other children, β = −0.50, *SE* = 0.14, *p* < 0.001 and to themselves, β = −1.24, *SE* = 1.18, *p* < 0.001. Children were also more likely to direct their questions to other children than to themselves, β = −0.74, *SE* = 1.17, *p* < 0.001. That is, as can be seen in Figure 3, questions took up a greater proportion (25%) of children’s adult-directed speech compared to the proportion of speech directed towards other children (17%) or themselves (9%). 

We then examined the effect of recipient type on linguistic properties of the questions. In each of the two mixed effects models predicting either the length or the diversity of words in questions with question type as a fixed effect and subject as a random effect, the effect of recipient type was not statistically significant (*p*s < 0.05). Thus, the length and lexical complexity of each question type did not differ by recipient type.

### 3.3. Examining the Recipient as a Function of Question Type

Of particular interest was whether the recipient of the questions varied as a function of question type (communication, information, and learning). To investigate this, we examined the proportion of each question type by recipient. Three mixed effects logistic regression models with recipient type as a fixed effect and subject as a random effect were fitted to predict the probability of questions intended for communication, information, and learning, respectively. In each model, the outcome variable was binary for the specific question category. For example, in a model that predicts the probability of questions intended for communication, the outcome variable was coded either 1 (communication) or 0 (non-learning including communication and information).

**Figure 4:**
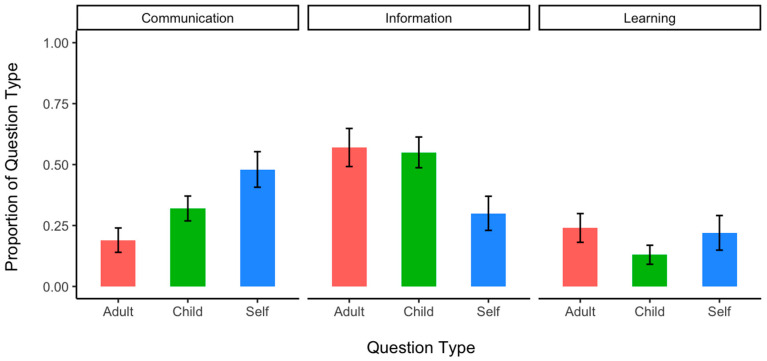
Proportion of questions intended for communication (**left**), information (**middle**), and learning (**right**) as a function of recipient. The proportion of questions relative to other speech acts based on the recipient of the speech was presented to account for the differences in the amount of speech directed to various recipients and the uneven child-to-adult ratio in each classroom. Error bars denote *SE*.

For the probability of questions intended for communication (Figure 4, left), children directed more communication questions to other children (32%), β = 0.92, *SE* = 0.36, *p* = 0.011 and themselves (48%), β = 1.31, *SE* = 0.43, *p* = 0.002 compared to adults (19%). There was no significant difference between the child and self categories in the likelihood of asking communication questions (32% vs. 48%), β = 0.39, *SE* = 0.36, *p* = 0.270. For the probability of questions intended for information (Figure 4, middle), none of the recipient type effects (57% adult vs. 55% child, 55% child vs. 30% self, 57% adult vs. 30% self) were statistically significant (*p* > 0.05).

For the probability of questions intended for learning (Figure 4, right), children directed significantly more learning questions to adults (24%) compared to other children (13%), β = −1.18, *SE* = 0.40, *p* = 0.003. (The patterns of the results were similar, but the effect becomes marginal after excluding children with their number of utterances beyond *M* ± 2*SD* (β = 0.87, *p* = 0.051) or the 95% quantile (β = 0.76, *p* = 0.071). All other results remained consistent after these exclusions.) There were no significant differences between the adult and self categories (24% vs. 22%), β = −0.99, *SE* = 0.54, *p* = 0.067, and between the child and self categories, (13% vs. 22%), β = 0.19, *SE* = 0.53, *p* = 0.722, in the likelihood of asking learning questions. Learning questions are the only type of question that consists of a greater proportion of adult-directed questions rather than child-directed questions.

We then examined the effect of question type and recipient type on the linguistic properties of the questions. We ran two mixed effects models predicting either the length or the diversity of words in questions with question type, recipient type, and their interaction as fixed effect factors and subject as a random effect. For the model predicting the length of questions in words, none of the interaction terms were statistically significant (*p* < 0.05). For the model predicting the diversity of words in questions such that lexical diversity was higher for information questions when asked to children than adults, β = 0.05, *SE* = 0.02, *p* = 0.042. However, follow-up analyses examining individual effects at specific levels of each moderating variable were not statistically significant, suggesting that the interactions may be present but the differences may not be strong or consistent enough to be considered statistically significant in isolation. Thus, the length and lexical complexity of each question type did not significantly differ by recipient type.

### 3.4. Examining Age Effects in Question Asking, Question Type, and the Recipient of Asked Questions

We next explored the degree to which question-asking strategies might differ across the preschool years by examining the effect of age. A mixed effects model predicting the proportion of questions with age as a fixed effect and subject as a random effect revealed no significant effect of age, β = −0.004, *SE* = 0.01, *p* = 0.699. In the mixed effects model predicting either the length questions in words with age and gender as fixed effects and subject as a random effect, the effect of age was not statistically significant, β = −0.01, *SE* = 0.03, *p* = 0.698. In the mixed effects model predicting diversity of words in questions, with age as a fixed effect and subject as a random effect, the effect of age was not statistically significant, β = −0.001, *SE* = 0.001, *p* = 0.110. Thus, the proportion of questions as well as the length and lexical complexity of questions did not differ by age.

We first focused on the role of age in the type of questions. To compare whether the type of questions differed by age, we fitted three mixed effects logistic regression models with age as a fixed effect and subject as a random effect to predict the probability of questions intended for communication, information, and learning, respectively. In each model, the outcome variable was binary for the specific question category (1: target category, 0: either one of the two non-target categories). Age was not a significant predictor of the likelihood of asking communication questions, β = 0.01, *SE* = 0.02, *p* = 0.549, information questions, β = −0.02, *SE* = 0.03, *p* = 0.576, or learning questions, β = −0.004, *SE* = 0.03, *p* = 0.893. Thus, the proportions of question types were similar regardless of age. We then examined whether the effects of question type on linguistic properties of the questions modified by age. In each of the two mixed effects models predicting either the length or the diversity of words in questions with question type, age, and their interaction as a fixed effect and subject as a random effect, neither the main effect of age nor the age × question type interaction was statistically significant (*p* < 0.05). Thus, the length and lexical complexity of each question type did not differ by age.

**Figure 5:**
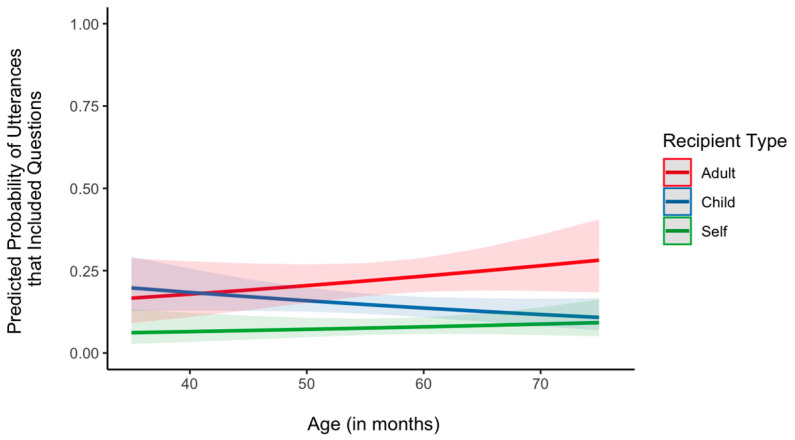
Predicted probability of utterances including questions as a function of age and recipient type. Shaded areas denote 95% CI.

Next, we examined the role of age in the type of recipients of asked questions, using mixed effects logistic regression models with recipient type as a fixed effect and subject as a random effect to predict the probability of questions (Figure 5). There was a significant interaction between age and child-directed questions, β = −0.03, *SE* = 0.02, *p* = 0.022. At the youngest age (37 months), children did not differ in the rate of questions directed to adults and other children, β = 0.13, *SE* = 0.32, *p* = 0.658. At the oldest age (76 months), however, children were less likely to direct their questions to other children than adults, β = −1.19, *SE* = 0.33, *p* = < 0.001. Lastly, the interaction between age and self-directed questions was not significant, β = −0.01, *p* = 0.766, but the main effect of self-directed questions was significant, β = 0.32, *p* = 0.010, indicating that children are less likely to direct their questions to themselves compared to adults regardless of age. Next, we compared the effects of recipient type on the linguistic properties of the questions modified by age. In each of the two mixed effects models predicting either the length or the diversity of words in questions with recipient type, age, and their interaction as fixed effect factors and subject as a random effect, neither the main effect of age nor the age × recipient type interaction was statistically significant (*p*s < 0.05). Thus, the length and lexical complexity of questions directed to each recipient was not modified by age. 

**Figure 6:**
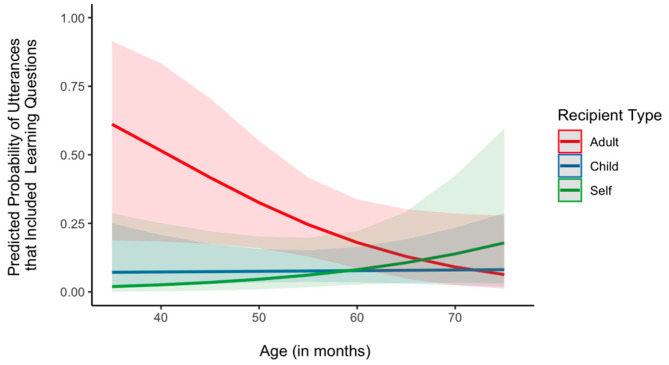
Predicted probability of utterances including learning questions as a function of age and recipient type. Shaded areas denote 95% CI.

Lastly, we examined the role of age in the association between recipient (adult, child, and self) and question types (communication, information, and learning). We fitted three mixed effects logistic regression models with recipient type, age, and their interaction as fixed effect factors and subject as a random effect to predict the probability of questions intended for communication, information, and learning, respectively. In each model, the outcome variable was binary for the specific question category (1: target category, 0: either one of the two non-target categories). The main effect of age and the age × recipient interaction did not significantly predict the likelihood of asking communication questions and information questions (*p* > 0.05). Thus, the proportions of question types were similar regardless of age. For learning questions, there was a significant interaction between age and the self-directed category, β = 0.14, *SE* = 0.06, *p* = 0.021. At the youngest age (37 months), children directed more learning questions to adults than to themselves, β = −4.11, *SE* = 1.55, *p* = 0.013. At the oldest age (76 months), however, children’s learning questions directed to adults and themselves did not differ, β = 1.31, *SE* = 1.10, *p* = 0.231 (Figure 6). The rest of the interactions were not statistically significant (*p*s < 0.05). We then examined whether the effects of question type and recipient on linguistic properties of the questions were modified by age. In each of the two mixed effects models predicting either the length or the diversity of words in questions with question type, recipient type, age, and their interaction terms as fixed effect factors and subject as a random effect, neither the main effect of age nor any interaction terms involving age were statistically significant (*p* < 0.05). Thus, the length and lexical complexity of each question type directed to different recipients were not modified by age. 

## 4. Discussion

Question asking is widespread in children’s spontaneous conversations. The findings of this study suggest that children are capable of using questions to actively seek out information from others in preschool environments. We found that preschoolers ask a higher proportion of questions when speaking to adults compared to when speaking to peers or themselves, particularly with questions that support broader learning goals. Notably, younger children tend to direct more learning-related questions to adults than their peers or themselves, whereas older children distribute their questions more evenly between adults and themselves, highlighting age-related differences in question-asking strategies. Despite using greater lexical diversity in their questions compared to other types of speech, the length and diversity of their questions remained consistent across different age and question types. In line with what we know about how question formulation trajectories develop in children, these results suggest that children are strategic in choosing both the content of their questions and whom to ask during everyday interactions. Our analysis from preschool settings offers a complementary perspective on previous work on question-asking behavior in controlled experiments and home settings.

While past work has looked at “wh-” questions (e.g., “what do you mean?” or “what is that?”) to indicate “learning” intent compared to other conversational speech, we adopted a novel and more nuanced approach to coding “wh-” questions. Specifically, we coded “wh-” questions as meeting one of three goals (communicative, informational, and learning) to tease apart the ambiguity in different meanings of the “wh-” questions. All goal types can fall under the category of “wh-” questions. If we were only to consider “wh-” questions (or only question length), then there would be insufficient or incorrect information to determine what the communicative goal of the question was. That is, some “wh” questions are not actually for learning in this deeper sense; for example, “what do you mean?” has a communicative goal, “what is your favorite color?” has an informational goal, while “what is that for?” has a learning goal. Furthermore, other non-”wh” questions have learning goals, such as “does this button make the machine go?”

Here, we developed a novel method for coding questions from children that capture one of these three intents in the “wh-” questions. This approach of analyzing the most common sentence structures for each question type has the benefit of enhancing specificity to identify question-asking patterns or outliers that may have been overlooked when grouped under a broader “wh-” category. While children can form questions with wh-words, rich syntactic structure, accurate dependency, etc., we also know that children can ask questions without syntactic marking by using simpler sentence structures. Thus, our coding of question types, coupled with the analysis of sentence structure, allows us to make a contribution of interest to applied and psycholinguistics by quantifying the linguistic complexity while coding the semantic intent. As a justification for this approach, we found that 17% of communication questions were short, using only one word as a “wh-word”. Additionally, 10% of information questions followed a pattern involving a “wh-word”, a verb, a preposition, and a noun phrase, supporting the idea that longer sentences with detailed descriptions were often used for seeking information. Furthermore, 8% of learning questions followed a structure of “wh-word”, a verb, and a determiner, indicating a fixed format used to aid learning. Thus, we suggest that future work in characterizing question-asking behavior might consider adopting this more semantic coding approach, although more laborious, as it better captures intention and uncovers a fuller picture of when questions are used in the learning sense.

Consistent with research on epistemic trust, we found that children exhibit the sensitivity to weigh information gains from different social partners. The proportion of questions in adult-directed speech was higher than those in child-directed and self-directed speech. Critically, children generated more questions with the intention of learning when talking to adults than to the other groups. Prior work has demonstrated that children can use principles such as information gain to effectively choose which questions to ask [55]. In our study, children directed more questions to adults than other children or themselves. In particular, adult-directed questions focused on content that is more likely to support general learning. These findings suggest that children are sensitive to who is informative and likely to provide helpful feedback. This initial result provides evidence to support the claim that children are not only being guided by these inferences about others [39,42], but are also actively using this knowledge to decide who to seek out for information.

Interestingly, a relatively large portion of children’s spontaneous speech, and questions in particular, were not just directed to other people, but rather self-directed. Self-directed questions stood for the lowest percentage out of total self-directed speech compared with adult- and child-directed speech. Moreover, self-directed questions were least used for learning, and more often for communicative and information purposes. For example, children often asked themselves communicative questions reflecting on an ongoing dialogue, e.g., “where?”. However, children also asked themselves detailed questions regarding a personal interest: “Why does she always throw these gummies in the bag?” While self-directed questions made up a similar percentage of all the questions asked in older and younger children, respectively, older children directed fewer learning questions to adults and more learning questions to themselves compared with younger children. When and why did they spontaneously ask questions of themselves during play and might this developmental effect take place? One hypothesis could be that older children are growing in autonomy and are engaging in self-explanation effects, articulating ideas aloud and working through them independently [56,57]. This phenomenon might also recruit cognitive processes that are more developed in older children, such as working memory and meta-cognitive monitoring [58]. Nevertheless, this finding that older children produce more self-directed speech contrasts with evidence that self-directed speech gradually becomes partially internalized between the ages of 3 and 5 [59]. Future work could explore how these factors, especially individual differences, typical or atypical development, and experiences with norms and guidance, condition these self-directed question-asking behaviors. For example, by using this study’s publicly shared database as a resource, future research can connect between the time-linked and linguistic characteristics of these questions and speech partner as self.

Our linguistic analysis revealed that preschoolers formulate questions similarly in terms of length and lexical diversity regardless of their age or the type of question recipient in preschool classrooms. This suggests that question formation is likely to reflect children’s general linguistic abilities, rather than being modified by social partners in their classrooms during the preschool years [5]. However, we found that children tended to use a greater variety of words when asking questions than in other utterances. Furthermore, learning questions contained a comparable number of diverse words to information questions, even though information questions had a higher word length. This suggests that it is worthwhile to further investigate the lexical diversity of children’s questions to gain a more comprehensive understanding of how they formulate questions.

Our data were collected during free play time, and there are potential contextual factors that may have contributed to children’s conversations. For example, the number of adults during free play was significantly lower compared to that of children. Thus, it is not surprising that the majority of the conversations were directed to other children. Furthermore, teachers may intentionally encourage children to communicate with other children during this period rather than engaging with children directly. We further explored whether speech partners provided explicit cues prompting children to ask questions (e.g., ”Do you have any questions for me?”, ”Go ask Mrs. [Teacher].”). However, only one of the 469 questions was preceded by these explicit cues. Thus, children appear to selectively orient their questions without direct guidance. Future work could explore whether the presence of implicit cues in the context such as pedagogical questions (e.g., “What are the colors of the rainbow?”) is related to the frequency and type of questions.

It is possible that other demographic or social factors may influence the quantity and content of questions. Previous studies showed that children ask fewer questions in schools when compared to home, which could be attributed to the naturalistic context of these studies, where speech was recorded over a time period where questions were not artificially elicited (as in an experiment using an active-learning-geared activity). Alternatively, such differences could be influenced by the social environments in schools, where children might rely on peers to ask questions or learn by observing others. Theories supporting school-based social norms, for example, in the US, suggest that children might be more sensitive to norms of answering questions rather than asking them in schools (for review, see [11,12,13]). We recognize that these norms may vary or may be culture- or school-specific, but this may explain why the older preschool children in our sample, who have likely had more exposure to these US school-based norms, may be more inclined to direct learning questions to themselves compared to adults in the school environment. Regarding socioeconomic status (SES), Kurkul and Corriveau (2018) [60] reported that low-SES children asked only half the number of questions to their caregivers compared to mid-SES children, despite no difference in question type. It would be an important next step to examine the extent to which socioeconomic backgrounds contribute to children’s question asking. In addition, when choosing from whom to learn, children tend to rely on an informant who shares the same culture or gender [43,44]. In our study, all of the teachers and researchers were female, but children’s questioning patterns did not differ by child’s gender. We think that future research that includes diverse speech partners can further explore the culture or gender match between children and their speech partners.

### Limitations and Future Directions

In this study, we analyzed children’s questions among preschoolers’ natural utterances in their classrooms. While mixed effects models used in this study are well suited for handling unbalanced data and smaller overall sample sizes, a larger sample size would increase the generalizability and robustness of our findings. Based on our robustness checks that compare results before and after excluding extreme cases, we found that, while all other results remained consistent, one finding became marginal after excluding children with the number of utterances beyond two standard deviations or the 95% quantile. Therefore, caution should be taken when generalizing the findings to broader populations or different contexts, as they are based on a relatively small number of children from a limited number of classrooms. Opportunities for children to ask questions in classrooms may also vary across cultures, educational norms and practices, and families, so future research should aim to include larger and more diverse samples and expand to different cultural and socioeconomic contexts to increase the robustness and generalizability of the findings from this study.

## 5. Conclusions

Many studies have explored children’s question asking in laboratory and controlled settings, but less work has explored how these social interactions support learning in more naturalistic settings and social contexts. Our research captures the opportunity to characterize and quantify children’s inquiries and interactions with different conversational partners with a novel and detailed linguistic analysis approach. In exploring such open social play windows sessions, where children spend many of their waking hours, we gain a richer understanding of the intricate social and educational dynamics within these learning environments. Children’s inquisitiveness is not just random, it is strategic. In everyday settings, our study shows that preschoolers adapt their questions based on the perceived expertise of their conversational partners. Our study also shows that these question-asking behaviors are nuanced in social settings; more complex, learning-oriented questions are directed towards adults, demonstrating discernment in both what to ask and whom to ask. Additionally, our data collection, including novel and diverse measures (e.g., question intent, type–token ratio), remains an open and valuable database for linguists and other researchers interested in the complex relationship between production types, conversational speakers, listeners, and social contexts. This work also clarifies important pedagogical opportunities; when teachers infer when and who children ask questions to, they may be able to capitalize on pedagogical windows to help children ready to learn. Thus, it remains an important area of study for our understanding of the environmental factors influencing early childhood development. In sum, when it comes to effective questioning, even young children understand that it is not just about the “what”, but also the “who”.

## Figures and Tables

**Table 1:** Examples of questions for each coding type.

Question Type	Example
Learning Question	“Do you know what this is?” “Is this a sponge?” “How do you write ‘F’?” * “Why?”
Communication Question	“The boy’s table?” “Wait, what (do) you mean?” “What is that again?” * “This?”
Information Question	“Can I get some water?” “Is my mom gonna be here soon? “Are you hungry? * “Where?”

Note. * Minimal length examples in each set.

## Data Availability

The original contributions presented in the study are included in the article, further inquiries can be directed to the corresponding authors.
